# Immune Response of Senegalese Sole against Betanodavirus Mutants with Modified Virulence

**DOI:** 10.3390/pathogens10111388

**Published:** 2021-10-27

**Authors:** Juan Gémez-Mata, Sandra Souto, Isabel Bandín, María del Carmen Alonso, Juan José Borrego, Alejandro Manuel Labella, Esther García-Rosado

**Affiliations:** 1 Instituto de Biotecnología y Desarrollo Azul (IBYDA), Departamento de Mi-Crobiología, Facultad de Ciencias, Universidad de Málaga, 29071 Málaga, Spain; juangemez@uma.es (J.G.-M.); mdalonso@uma.es (M.d.C.A.); jjborrego@uma.es (J.J.B.); amlabella@uma.es (A.M.L.); 2 Instituto de Acuicultura, Departamento de Microbiología y Parasitología, Universidade de Santiago de Compostela, 15782 Santiago de Compostela, Spain; sandra.souto@usc.es (S.S.); isabel.bandin@usc.es (I.B.)

**Keywords:** *Solea senegalensis*, reassortant nervous necrosis virus, 3’ non-coding region, immune response, OpenArray^®^

## Abstract

Nervous necrosis virus (NNV), genus *Betanodavirus*, the etiological agent of the viral encephalopathy and retinopathy (VER), presents a genome with two positive-sense single-stranded RNA segments. Striped jack nervous necrosis virus (SJNNV) and red-spotted grouper nervous necrosis virus (RGNNV), together with reassortants RGNNV/SJNNV, are the betanodaviruses predominantly isolated in Southern Europe. An RGNNV/SJNNV reassortant isolated from Senegalese sole (wt160) causes high mortalities in this fish species. This virus presents differences in the sequence of the 3’ non-coding region (NCR) of both segments compared to RGNNV and SJNNV reference strains. Previously, it has been reported that the reversion of two of these differences (nucleotides 1408 and 1412) in the RNA2 3’NCR to the SJNNV-type (recombinant r1408-1412) resulted in a decrease in sole mortality. In the present study, we have applied an OpenArray^®^ to analyse the involvement of sole immune response in the virulence of several recombinants: the r1408-1412 and two recombinants, developed in the present study, harbouring mutations at positions 3073 and 3093 of RNA1 3’NCR to revert them to RGNNV-type. According to the correlation values and to the number of expressed genes, the infection with the RNA2-mutant provoked the most different immune response compared to the immune response triggered after the infection with the rest of the viruses, and the exclusive and high upregulation of genes related to the complement system. The infection with the RNA1-mutants also provoked a decrease in mortality and their replication was delayed at least 24 h compared to the wt160 replication, which could provoke the lag observed in the immune response. Furthermore, the infection with the RNA1-mutants provoked the exclusive expression of *pkr* and the downregulation of *il17rc*.

## 1. Introduction

Viral nervous necrosis (VNN), also called viral encephalopathy and retinopathy (VER), is a viral disease affecting a high number of fish species of great interest in aquaculture, such as sea bass (*Dicentrarchus labrax*), Senegalese sole (*Solea senegalensis*) and gilthead seabream (*Sparus aurata*) [1]. The etiological agent of this disease is the nervous necrosis virus (NNV), which belongs to the *Nodaviridae* family, *Betanodavirus* genus. NNV is a naked virus and presents bisegmented positive-sense single-stranded RNA. RNA1 encodes the RNA-dependent RNA polymerase (RdRp), whereas RNA2 encodes the capsid protein (CP). According to a variable region in the RNA2 segment (named T4), NNV has been classified into four species: striped jack nervous necrosis virus (SJNNV), tiger puffer nervous necrosis virus (TPNNV), red-spotted grouper nervous necrosis virus (RGNNV), and barfin flounder nervous necrosis virus (BFNNV) [2]. Furthermore, reassortants presenting combinations of RGNNV and SJNNV segments have been isolated from different fish species [3,4,5,6,7]. Thus, Olveira et al. [4] reported the isolation of a reassortant from Senegalese sole, named SpSsIAusc160.03 (hereafter wt160), with a genome composed of RGNNV-type RNA1 and SJNNV-type RNA2 segments (RGNNV/SJNNV). This reassortant provokes high mortality in this fish species and exhibits differences in the 3’ non-coding region (NCR) of both segments when compared to the reference strains of each species [4,8]. Regarding RNA1, four different positions were observed, whereas five differences were found in the RNA2 [8]. Two of the differences observed in the 3’NCR of the RNA2 are located at positions 1408 and 1412, sited in a stem-loop structure (3’SL), which is essential for replication [9]. Their reversion to SJNNV-type nucleic acids resulted in ca. 70% decrease in sole mortality compared to the wild type virus. This effect on the virulence has been attributed either to an impaired interaction of RNA2 with host proteins, or to a differential immune response [9].

On the other hand, the effect on virulence of the above-described differences in the RNA1 has not been determined. Therefore, in the present study, we have examined the effect of two of these changes (position 3073 and 3093) on virulence by the development of two recombinant viruses harbouring point mutations, which make the RNA1 similar to the reference RGNNV virus. Furthermore, in order to determine the involvement of the fish immune response in betanodavirus virulence to sole, the 112 OpenArray^®^ designed by Gémez-Mata et al. [10] has been applied to analyse the immune gene expression after the infection with the recombinant viruses harbouring mutations at the 3’NCR of RNA1 or RNA2.

## 2. Results

### 2.1. Cumulative Mortality and Viral Replication

**Figure 1:**
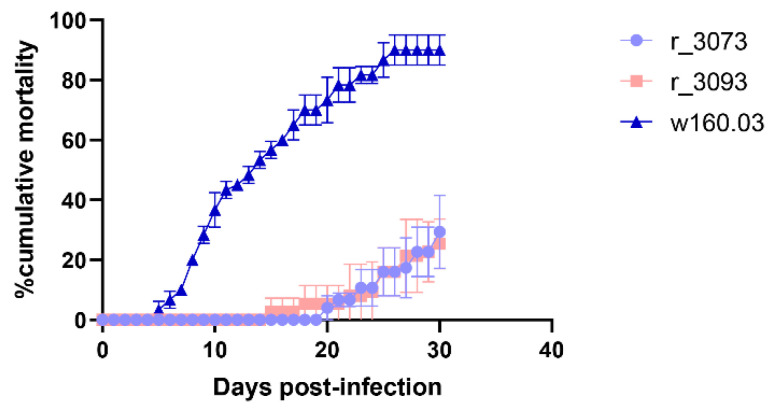
Accumulated mortality after the infection with the wild type virus wt160 and the mutants r3073 and r3093. Fish (*n* = 30/tank) were bath challenged over a period of 3 h in sea water containing 10^5^ TCID_50_/mL of each viral strain. The curves represent the mean of three different experiments ± SD. Data were analysed with the Kaplan-Meyer test.

The challenge of Senegalese sole with wt160 resulted in 90% cumulative mortality at 30 days post-infection (p.i.), whereas the infection with r3073 or r3093 (RNA1 mutants) decreased the mortality percentages to 29.3% and 25.3%, respectively. In addition, a delay in the mortality onset was observed (Figure 1). 

Thus, in the wt160-infected group mortality was firstly recorded at 5 days p.i.; whereas in the r3073 and r3093 groups mortality onset was at 15 and 20 days p.i., respectively.

**Figure 2:**
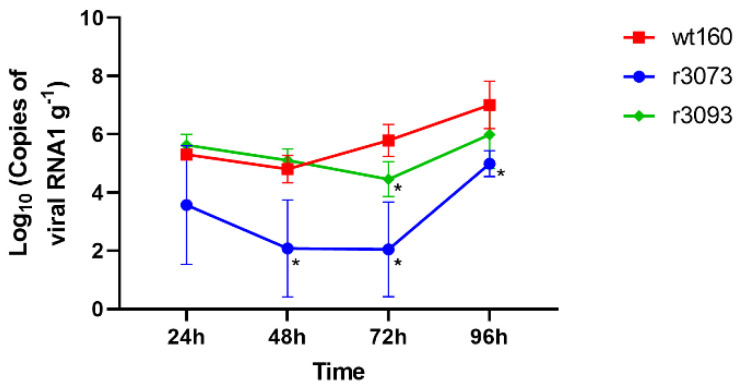
RNA replication in sole brain. The number of genome copies in brain tissue was determined by RT-qPCR. The results were expressed as the mean ± SD (*n* = 6). * *p* < 0.05 compared to wt160 at the same sampling time using Student t test analysis adjusted by the Bonferroni method for multiple comparison using GraphPad Prism.

The analysis of the viral genome in randomly sampled fish showed a significant increase in the number of wt160-RNA1 copies from 48 to 96 h p.i. (Figure 2). 

The number of r3093-RNA1 copies at 24 and 48 h p.i. was similar to that obtained for the wt160 group at the same times p.i.; however, at 72 h p.i., quantification of r3093 RNA1 copy number reached values significantly lower than those obtained for wt160 (Figure 2). Replication of this recombinant virus seems to be 24-h delayed compared to the wild type. Thus, whereas an increase in wt160 replication was observed from 42 to 72 h p.i., the increase in r3093 replication was recorded 24 h later, from 72 to 96 h p.i., time point when RNA1 reached values slightly lower than those reported for wt160, although no significant differences were recorded (Figure 2).

On the other hand, brains from fish infected with r3073 displayed the lowest values of RNA1 copies at all time points tested. Although an increase in viral genome copy number was also observed in this experimental group from 72 to 96 h p.i., final values were significantly lower than those obtained for the wt160 (Figure 2). 

### 2.2. Overview of DEGs after Viral Infection

**Figure 3:**
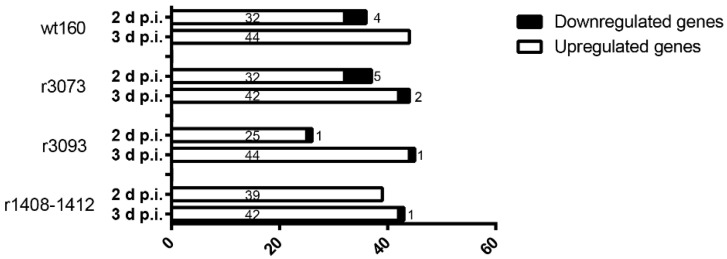
Total number of differentially expressed genes in the head kidney of Senegalese sole sampled 2 and 3 days after the infection with the wild type virus wt160 and the mutants r1408-1412, r3073 and r3093. Upregulated genes appear in white, downregulated genes are in black.

In general terms, the number of DEGs in head kidney samples was quite similar in all virus-infected groups, being higher at 3 than at 2 days p.i. (Figure 3). 

Most of DEGs were upregulated; specifically, at 2 days p.i., the number of DEGs ranged from 26 (25 upregulated and 1 downregulated) in the r3093-infected group (RNA1 mutant) to 39 (all of them upregulated) after the infection with the double-RNA2 mutant (r1408-1412), with 36 (32 upregulated and 4 downregulated) as the number of DEGs recorded in the wt160-challenged group and 37 after infection with r3073, 32 upregulated coinciding with the number of upregulated genes after the infection with the wt160 at that time point.

At 3 days p.i., the number of DEGs ranged from 43 (42 upregulated and 1 downregulated) after r1408-1412 infection to 45 (44 upregulated and 1 downregulated) in the r3093 group (Figure 3). At that sampling time, the number of DEGs in the wt160 group was 44 (all of them upregulated), coinciding with the number of DEGs after r3073 infection (RNA1 mutant) (42 upregulated and 2 downregulated) (Figure 3). The level of expression of immuno-related genes in the head kidney of Senegalese sole infected with the four viruses at both sampling times is represented in Table S1. 

**Figure 4:**
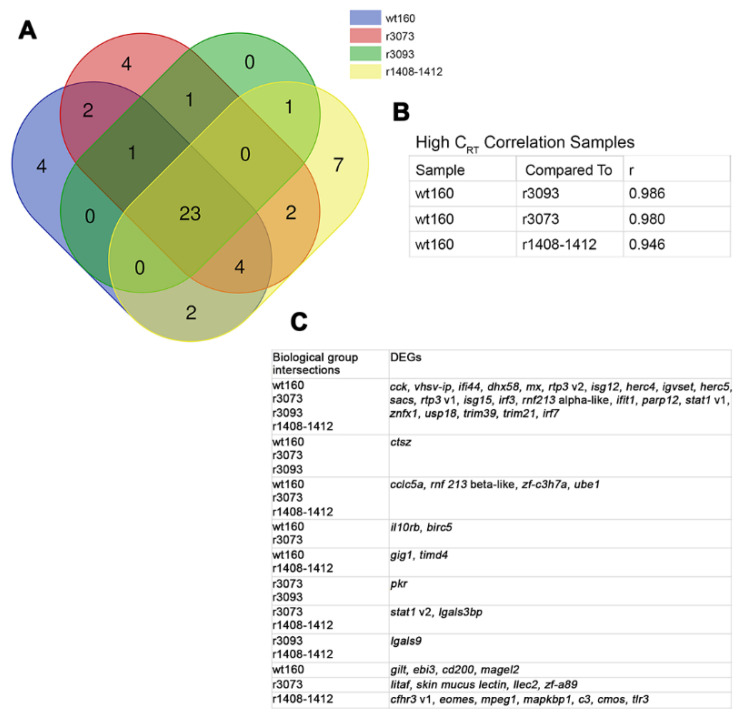
Differentially expressed genes (DEGs) in *Solea senegalensis* 2 days after the infection with wt160, r1408-1412, r3073 or r3093. (**A**) Venn diagram analysis of the number of DEGs shared by the four virus-infected groups. (**B**) Correlation coefficients (r) of target gene Cq values obtained after the infection with each mutant confronted with values obtained after the infection with the wild type virus. (**C**) Gene profile of each overlapping area obtained with the Venn diagram analysis.

**Figure 5:**
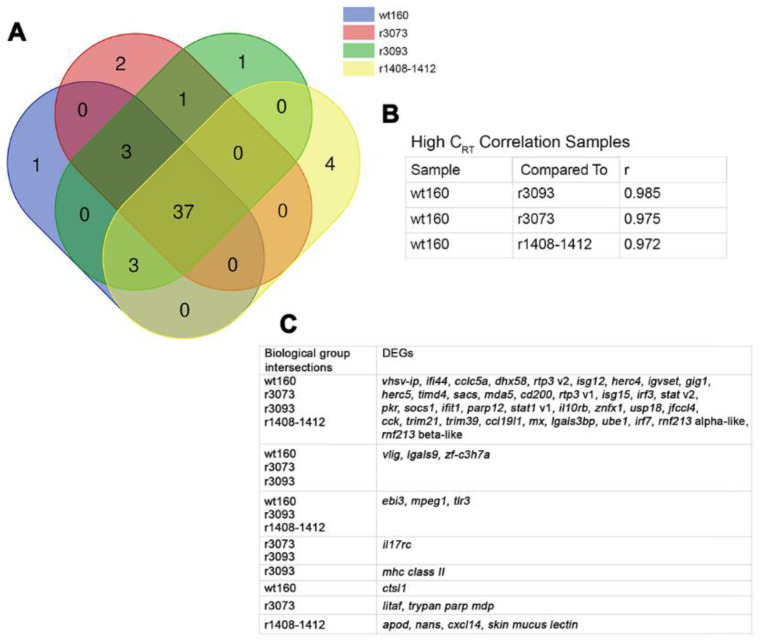
Differentially expressed genes (DEGs) in *Solea senegalensis* 3 days after the infection with wt160, r1408-1412, r3073 or r3093. (**A**) Venn diagram analysis of the number of DEGs shared by the four virus-infected groups. (**B**) Correlation coefficients (r) of target gene Cq values obtained after the infection with each mutant confronted with values obtained after the infection with the wild type virus. (**C**) Gene profile of each overlapping area obtained with the Venn diagram analysis.

The Venn diagram method was used for the comparative analysis of DEG datasets obtained 2 and 3 days after the infection with all the viruses considered (Figure 4A and Figure 5A, respectively) (Bioinformatics and Evolutionary Genomics; http://bioinformatics.psb.ugent.be/webtools/Venn/ accessed on 24 May 2021). According to these diagrams, the number of deregulated DEGs shared by all virus-infected groups was lower at 2 days (23) than at 3 days p.i. (37) (Figure 4A and Figure 5A). The correlation coefficients were obtained by confronting fold change values recorded in fish infected with each mutated virus and in wt160-challenged fish were calculated using the RQ software (Applied Biosystems^TM^, Carlsbad, CA, USA). According to this analysis, the infection with the RNA2-double mutant induced the most differentiated immune response at both time points (r value: 0.946 at 2 days p.i. and 0.972 at 3 days p.i.), whereas the highest correlation coefficient was recorded for the r3093 group at both time points (r value: 0.986 at 2 days p.i. and 0.985 at 3 days p.i.) (Figure 4B and Figure 5B). 

Most of the genes expressed in all virus-infected groups were related to the interferon (IFN)-I pathway (viral recognition, regulation of IFN-I-dependent immune response, JAK-STAT cascade and IFN-stimulated genes), virus response genes (VRGs) and protein ubiquitination (Figure 4C and Figure 5C).

**Figure 6:**
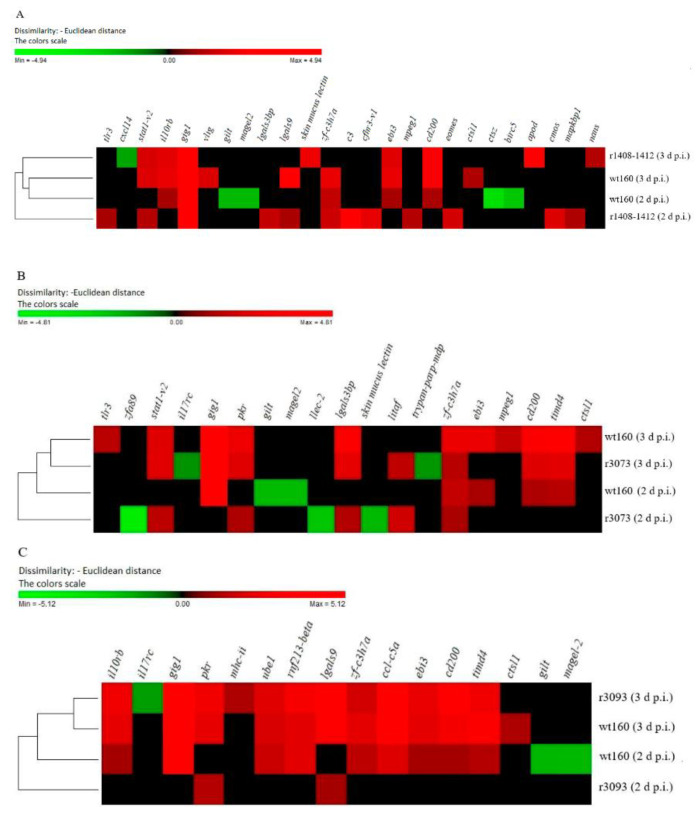
Hierarchical clustering analysis based on set of differentially expressed transcripts in the head kidney of Senegalese sole infected with wt160 or r1408-1412 (**A**); r3073 (**B**) or r3093 (**C**). Samples were collected at 2 and 3 days p.i. Data are expressed as log_2_ of fold change. Green and red colours indicate down and upregulated genes according to the scale shown.

The clustering analyses of genes differentially transcribed after the infection with each mutant, compared to DEGs recorded in the wt160 group, are represented in Figure 6. 

In this regard, it should be highlighted those genes that were only deregulated after the infection with the most virulent virus, wt160. For instance, gamma-interferon-responsive lysosomal thiol gene (*gilt*) and melanoma-associated gene (*magel2*) were downregulated at 2 days p.i.; whereas the upregulated genes were *ebi3* and *cd200* at 2 days p.i. (Figure 4C and Figure 6) and *ctsl1* at 3 days p.i. (Figure 5C and Figure 6).

### 2.3. DEG Profiling after RNA2-Mutant Infection, r1408-1412

As mentioned above, according to the correlation values and to the number of genes exclusively expressed after the infection with the RNA2-double mutant (7 at 2 d p.i. and 4 at 3 d p.i., Figure 4A and Figure 5A), this virus triggered the most differentiated immune response, both at 2 and 3 days p.i. Specifically, the highest differences between wt160- and r1408-1412-infected fish were detected at 2 days p.i. (r value: 0.946; Figure 4B). At that sampling time, 10 genes were exclusively expressed in animals infected with the double mutant (Figure 4A and Figure 6A). Regarding these genes, it should be highlighted *c3* and *cfhr3* (involved in the complement system), and *eomes* related to the regulation of T-cell proliferation (Figure 6A).

At 3 days p.i., four genes were exclusively expressed after the infection with the double mutant (Figure 5A and Figure 6A). Specifically, the infection with r1408-1412 upregulated the expression of a skin mucus lectin, a gene codifying for an apolipoprotein D, *apoD*, and a VRG, *nans*. Furthermore, the gene coding for a CXC chemokine, *cxcl14*, was downregulated exclusively as a consequence of the double mutant infection (Figure 6A). 

### 2.4. DEG Profiling after Infection with RNA1 Mutants, r3073 and r3093

Although the expression of IFN-I-related genes was quite similar for the four viruses considered (Figure 4C and Figure 5C), there were differences in the pattern expression of three specific IFN-I-related genes in fish infected with both RNA1-mutated viruses. Thus, *pkr* was induced 2 days after infection with both RNA1-mutants, but it was not transcribed in fish from the wt160-infected group neither in the r1408-1412-infected group (Figure 4C and Figure 6). On the other hand, at 3 days p.i., *il17rc*, involved in the JAK-STAT cascade, was only downregulated after the infection with both RNA1-mutants (Figure 5C and Figure 6). Finally, *gig1* was not transcribed 2 days after the infection with r3073 or r3093, although it was expressed at the highest level after the infection with the four viruses at 3 days p.i. (Figure 6).

Regarding the infection with r3073, this virus downregulated the expression of some VRGs at 2 days p.i., such as *llec-2*, a skin mucus lectin, and *zfa89*, related to viral recognition. At 3 days p.i., only three genes were expressed significantly different after r3073 infection compared to the infection with wt160 (Figure 5A and Figure 6B).

The infection with r3093 resulted in the deregulation of 26 DEGs at 2 days p.i., as is represented in Figure 3; however, most of them with fold change values similar to those recorded in the wt160-infected group (Figure 6C). Specifically, *pkr* and *lgals9* were significantly upregulated in the r3093-infected group, compared to the wt160-infected group, at 2 days p.i. (Figure 6C). Furthermore, the expression pattern at 3 days p.i. is quite similar to that observed after the infection with the wild type at the same sampling time (Figure 6C).

## 3. Discussion

In this study, the transcription of Senegalese sole immune genes triggered by reassortant betanodaviruses presenting different mutations in the 3’NCR of both genomic segments has been quantified. The stability of the mutations was confirmed by sequencing at the end of the experimental challenges. The transcriptomic study has been performed using an OpenArray^®^ platform previously designed [10]. In general, the total number of deregulated DEGs after the infection with all the viruses has been similar; most of these genes were upregulated, with the total number of DEGs slightly higher at 3 than at 2 days p.i. This result is not consistent with the results previously obtained after wt160-intramuscular injection [10], which induced a higher number of DEGs at 2 than at 3 days p.i. in the head kidney. However, it should be considered that the viral administration in both studies was different. According to Souto et al. [11], at the initial phase of the mortality curve, the titre of wt160 in brains from intramuscularly injected sole was higher (1.2 × 10^9^ TCID_50_/g) than in bath-challenged fish (1.3 × 10^5^ TCID_50_/g), which could stand for an earlier immune response after the intramuscular injection. In fact, in the present study, at 2 days p.i., we have recorded the downregulation of certain genes non-deregulated after intramuscular injection (*gilt*, *magel2*, *ctsz* and *birc5*) [10] (Figure 6). According to these results, the downregulation of these genes after wt160 intramuscular injection could have taken place earlier. In fact, recently, Peruzza et al. [12] has also described the downregulation of immune genes after the infection of gilthead seabream larvae with a RGNNV/SJNNV strain at the initial phase of the infection (6 and 12 h p.i.), suggesting that his mechanism favours the immune evasion by the virus

Specifically, in the present study, *gilt* and *magel2* were exclusively downregulated after the infection with the most virulent virus, the wild type strain. In mammals, the gamma-interferon-inducible lysosomal thiol reductase (GILT) is involved in the processing and presentation of antigens into the major histocompatibility complex (MHC) class II [13]. Similar functions have also been described for several orthologous fish genes [14,15,16,17,18,19,20,21]. Furthermore, it has been described that GILT controls the replication of murine leukaemia virus and human immunodeficiency virus type 1 [22,23]; therefore, the *gilt* downregulation detected in wt160-infected animals could contribute to increase viral virulence. 

Regarding *magel2*, this gene encodes the melanoma-antigen-subfamily-like-2 protein (MAGEL2), belonging to the necdin/MAGE gene family. MAGEL2 is part of a large ubiquitination complex that regulates endocytosis, receptor recycling and cell-surface localization [24]; furthermore, data suggest that it is involved in the regulation of cell cycle progression and apoptosis restriction [25]. Its role during viral infection is unknown; however, its capacity to regulate endocytosis could be indirectly implied in the control of the viral entrance into the cells. 

Infection with the RNA2-double mutant elicited the most differentiated immune response, compared to the wt160 group, at both time points (Figure 4, Figure 5 and Figure 6A). According to Souto et al. [9], the mutations in the 3’NCR of wt160 RNA2 produced a disruption of the 3’SL structure, which would be responsible for the increase in the survival rate of infected soles. However, the disruption of the 3’SL did not affect viral replication, at least at 2 and 3 days p.i., although a significant decrease in the number of RNA1 and RNA2 copies was observed at 4 days p.i. [9]. Therefore, the differentiated immune response detected in the present study after the infection with the RNA2-mutant could be responsible for the decrease in replication and, therefore, for the decrease of mortality described by Souto et al. [9], although further assays should be performed to confirm this hypothesis. 

Focusing on specific routes of the immune response, it is worth highlighting the exclusive deregulation of the complement pathway after the infection with the double RNA2 mutant, upregulating *c3* and the regulator of the complement cascade *cfhr3* only at 2 days p.i. The early upregulation of *c3* has been previously described in gilthead seabream and European seabass infected with betanodavirus [26]. The results obtained for seabass are similar to those described in the present study, *c3* expression returns to basal levels at 3 days p.i. [26], whereas in gilthead seabream, the overexpression of *c3* remained high for longer [26]. On the other hand, in humans CFHR3 has been described as an enhancer of the complement activation, competing with the inhibitory complement factor H, and enhancing the formation of C3 convertase, resulting in C3bi, a subproduct of C3b, an inductor of phagocytosis and inflammation [27]. The upregulation of both *c3* and *cfhr3* could partly explain the lower mortality recorded by the double mutant infection through the induction of an early inflammation response, which, due to the return to basal level of expression of both complement factors at 3 days p.i., is inefficient for controlling viral replication.

In addition, another two genes that were only deregulated after infection with the double mutant were *eomes* at 2 days p.i., and, at 3 days p.i., *apoD*, which reaches one of the highest expression levels (3.94). The gene *eomes* is related to CD8^+^ T-cell differentiation. The involvement of T-cells in the immune response against nodavirus infection has been previously reported in Atlantic halibut (*Hippoglossus hippoglossus* L.) by Øvergard et al. [28], who described the upregulation of CD8α and CD8β transcripts in the brains of challenged fish. On the other hand, *apoD* encodes a lipocalin, which has been described in higher vertebrates as a protector of neurological disorders, controlling peroxidated lipids [29] and regulating inflammatory processes involved in neuronal damage [30]. In addition, it has been reported that *apoD* is upregulated in mice during acute encephalitis induced by the human coronavirus OC43 (HCoV-OC43) and produced T-cell infiltration into the brain, which is correlated to an increase in mouse survival rate [31]. Therefore, the upregulation of *eomes* and *apoD* recorded in the head kidney of r1408-1412-infected fish could indicate a systemic immune response to protect Senegalese sole from neuronal damages, reducing inflammation, and, therefore, could be related to the decrease in mortality observed after the infection with the double mutant. 

Regarding RNA1-mutated viruses, the mutations in the 3’NCR of this segment reduced the mortality to 29,3% and 25,3%, for r3073 and r3093, respectively. Mutations also provoked some differences in viral replication—the number of copies of r3073 RNA1 was lower than that recorded for the wt160-infected group at all time points assayed. However, this result did not cause a reduced immune response, since the number of deregulated genes after the infection with both viruses, r3073 and wt160, was similar. This result suggests that a low amount of r3073 RNA is enough to induce an efficient immune response in fish. Similar results have been previously reported for the infection of sole with a non-pathogenic viral haemorrhagic septicaemia virus (VHSV) isolated to this fish species [32]. Regarding the replication of r3093, a 24 h lag was observed compared to wt160 replication. This delay seems to provoke a lag in the immune response, since r3093 infection deregulates the lowest number of genes at 2 days p.i. (25 upregulated and 1 downregulated) (Figure 3), whereas the number of deregulated genes 3 days after the infection with this virus was similar to that recorded in the other infected groups. 

However, regarding specific genes, infection with the RNA1-mutated viruses provoked a differentiated immune response, which could be partly responsible for the decrease in mortality, hence the exclusive *pkr* upregulation at 2 days p.i., and *il17rc* downregulation at 3 days p.i., as well as an inability to induce *gig1* at 2 days p.i. However, the expression of this gene reached the highest level at 3 days p.i.

Regarding *pkr*, although the double-strand RNA (dsRNA)-dependent protein kinase receptor (PKR) has been designed as an IFN-stimulated gene (ISG), this protein is also able to directly recognize viral dsRNA, with high affinity, or single-stranded RNA (ssRNA), with lower affinity, through an IFN-independent mechanism, acting as a pattern recognition receptor (PRR) [33,34]. Therefore, the RNA1-mutant genome could be detected by PKR at the beginning of the infection, even before the stimulation of the IFN-I system, and, therefore, could collaborate in the protection against these infections. 

In addition, *il17rc* encodes an interleukin receptor, which binds the proinflammatory cytokines IL-17A and IL-17F [35]. Therefore, the downregulation of *il17rc* later in the infection by RNA1-mutants could reduce inflammation, one of the main factors involved in the severity of the disease [36]. Furthermore, the downregulation of this gene has also been described by Tuipulotu et al. [37] after the infection of macrophages with a murine-norovirus, where, together with other downregulated innate receptors, *il17rc* seems to be implied in counteracting the immediate viral recognition and in the induction of early antiviral response, dampening host defences. Therefore, *il17rc* downregulation only after infection with RNA1-mutants could counteract the early immune response, preventing an increase in the disease severity due to a strong immune response.

Finally, it should be highlighted that, although *gig1* was not transcribed 2 days after the infection with r3073 and r3093, this gene was expressed with the highest fold change value 3 days p.i. in all experimental groups (Figure 6). The grass carp reovirus (GCRV)-induced gene 1 was detected in UV-inactivated GCRV-infected crucian carp (*Carassius auratus*) blastulae embryonic cells (CAB) [38], as well as in Atlantic salmon (*Salmo salar*) and zebrafish (*Danio rerio*) [39,40,41]. Sun et al. [42] reported that *gig1* is an ISG that only exits in fish lineages, and that GIG1 protein suppressed GCRV replication in cultured fish cells. According to the results obtained in the present study, it seems that an early expression of this gene, together with the expression of other interferon-related genes, does not control viral infection; however, a constrained expression seems to decrease virulence. This hypothesis is related to the idea previously reported by several authors, suggesting that a strong and early immune response, rather than the control of betanodavirus infection, is responsible for the aggravation of the disease [10,43,44]. 

## 4. Materials and Methods

### 4.1. Cell Cultures and Viruses

E-11 cells [45] were maintained in Leibovitz L-15 medium (Gibco; Life Technologies Limited Paisley, Renfrew, UK) supplemented with 5% foetal bovine serum (FBS) and 2 mM L-glutamine (Lonza; Verviers, Belgium) at 25 °C. BSRT7/5 cells kindly provided by Dr K. K. Conzelmann (Ludwig-Maximilians-Universitat Munich) [46], were maintained in Dulbecco’s modified Eagle’s medium (DMEM; Lonza) supplemented with 10% FBS, 2mM L-glutamine, penicillin (100 units mL^−1^) and streptomycin (100 mg mL^−1^) at 37 °C in a 5% CO_2_ humidified chamber. Geneticin (G418, 1 mg mL^−1^ final concentration, Gibco) was added every two subcultures.

The following recombinant viruses, developed from the wt160 sequence, have been used in this study: r1408-1412, containing the nucleotide changes T1408C and A1412T in the RNA2 3’NCR [9]; r3073, harbouring the C3073T mutation in the RNA1 3’NCR; and r3093, with the mutation T3093C in the RNA1 3’NCR. The wild type RGNNV/SJNNV wt160 (isolated from sole [4]) has also been used for comparative purposes. Viral strains were propagated on E-11 in L-15 medium supplemented with 2% FBS, 100 IU mL-1 penicillin and 10 mg mL-1 streptomycin and incubated at 25 °C until the appearance of cytopathic effect (CPE).

Recombinants r3073 and r3093 were obtained by reverse genetics as previously described [4,9]. Briefly, the plasmid pBS160R1 [8], which contains the full-length cDNA of the wt160 RNA1 sequence, was subjected to site-directed mutagenesis using the QuickChange Multi Site-Directed Mutagenesis kit (Agilent Technologies; West Cedar Creek, TX, USA) using the specific primer MutR1end3073-5’ TTTGGTCCCTTAATCAGCTTTATGCTGTCTTACGCTTCGG3’ and MutR1end3093- 5’TTTGGTCCCCTAATCAGCTTTATGCTGTCCTACGCTTCGG3’. Plasmids obtained are pBS160R1_3073 and pBS160R1_3093. In order to generate recombinant viral particles, BSRT7/5 monolayers [46] were transfected with a 1:1 mixture of RNA2 (pBS160R2, [8]) and RNA1 (either pBS160R1_3073 or pBS160R1_3093). This transfection was conducted with the Lipofectamine2000 reagent (Invitrogen; Life Technologies Co., Carlsbad, CA, USA) according to the supplier’s instructions. Cells were incubated for 12 h at 37 °C and then shifted to 28 °C for 7 days with DMEM containing 2% FBS. Afterwards, cells were collected by scraping, and then subjected to freezing/thawing. Cellular debris was then clarified by centrifugation, and supernatants were subjected to several passages on E-11 cells until the appearance of CPE. The presence of the mutations in the recovered viral particles was confirmed by sequencing (GATC Biotech; Ebersberg, Germany).

Viral titration was performed on E-11 cells growth in 96-well plates incubated at 25 °C for 7–10 days. Cells were examined daily for CPE observation, and titres, expressed as TCID50 mL-1, were calculated according to the end-point dilution method [47].

### 4.2. Experimental Challenges

Experimental infections were performed to determine the virulence of the 3’NCR-RNA1 mutants (r3073 and r3093), and to analyse the immune response against the three recombinants (r1408-1412; r3073 and r3093) in comparison with the immune gene transcription in fish infected with the wild type (wt160).

Juvenile sole (mean weight 2 g) were obtained from a commercial fish farm and maintained at the fish facilities of the University of Santiago de Compostela (Spain). Fish were handled in strict accordance with good animal practices as defined by the European Union guidelines for the handling of laboratory animals (directive 2010/63/UE). The protocol was approved by the Galician Committee for experimental animal welfare and the Xunta de Galicia (Permit Id. 15010/2020/004). Fish were fed daily ad libitum with dry commercial pellets (Skretting Gemma 1.2, Skretting; Burgos, Spain). They were acclimatised for 10 days in opaque 100 L-tanks at 22 °C. Before experimental infection, 5 fish were sacrificed with an anesthetic overdose (MS-222, Sigma-Aldrich, St. Louis, MO, USA) and used for the diagnosis of bacterial and viral pathogens as described by Olveira et al. [48].

Four groups of juvenile sole (*n* = 80) were infected with each of the three mutants or with the wild type strain. In addition, a fifth control group (*n* = 20) was also set up using L-15 medium as mock infection. All challenges were performed by bath at a virus concentration of 10^5^ TCID_50_ mL^−1^ for 3 h, with strong aeration. Afterwards, animals were maintained at 22 °C and euthanized by MS-222 overdose at different time points p.i. In order to study the immune response, individual samples of head kidney were aseptically recovered from six fish per experimental group at 2 and 3 days p.i. Samples were frozen in liquid nitrogen and stored at −80 °C until used. The immune response was analysed using an OpenArray^®^ platform previously designed [10]. On the other hand, six fish from each tank were sampled at 24, 48, 72 and 96 h p.i. and brain tissues were analysed individually by RT-qPCR [48] to quantify viral RNA. In parallel, three groups (*n* = 30) per condition, were challenged as described above to calculate mortality provoked by r3073, r3093 and wt160. At the end of the experimental infections, the maintenance of mutations in each recombinant strain was confirmed by sequencing (GATC Biotech, Germany).

### 4.3. RNA Extraction and cDNA Synthesis

To determine viral replication, brains were directly homogenized with lysis solution and total RNA was extracted using the Nucleospin ^®^ Kit (Macherey-Nagel; Düren Germany) following manufacturer´s instructions. Extracted RNA was reverse transcribed with the Superscript IV reverse transcriptase (Invitrogen; Waltham, MA, USA) using random primers. 

Head-kidney samples were homogenized in 1 mL of TRI Reagent (Sigma-Aldrich; San Luis, MO, USA) using the MM400 (Retsch; Haan, Germany) homogenizer. A volume of 100 µL of 1-bromo-3-chloropropane (AppliChem; Darmstadt Germany) was added, and samples were centrifuged at 12,000× *g* at 4 °C for 5 min. The aqueous phase was recovered, and an equal volume of 75% ethanol was added. RNA extraction was carried out using the RNeasy Mini Kit (Qiagen; Düsseldorf, Germany) following manufacturer’s instructions. RNA was quantified by spectrophotometry at 260 nm using the NanoDrop system (ThermoFisher Scientific; Waltham, MA, USA). RNA quality was determined by the absorbance ratios A_260/230,_ between 2.0 and 2.4, and A_260/280_, between 1.8 and 2.1, as well as by the RNA integrity number (RIN), between 8 and 10. cDNA was synthetized using MicroAmp Optical 96-well reaction plates (Applied Biosystems^TM^) and the High-Capacity cDNA Reverse Transcription Kit (Applied Biosystems^TM^). RNA (2 µg) was added to each well containing 2 µL of 10X RT Buffer, 2 µL of 10X RT Random Primers, 1 µL of 25X dNTPs, 1 µL of MultiScribe^TM^ Reverse Transcriptase and 4 µL of RNase-free water. The synthesis profile was 10 min at room temperature, 2 h at 37 °C, 5 min on ice, 10 min at 75 °C and 5 min on ice.

### 4.4. Quantification of Viral RNA 

qPCR reactions were set up with 2 µL of cDNA in 20 µL final volume, using iQTM SYBR^®^ Green Supermix (Bio-Rad, Hercules, CA, USA) and 200 nM of primers SnodR1 F/R [48]. Quantification of genome copies was performed using a standard curve consisting of 20-fold dilutions (10^1^–10^7^ copies mL^−1^) of a DNA plasmid containing RNA1 full-segment of wt160. Reactions were carried out in a CFX96TM Real-Time PCR Detection System (Bio-Rad); following an initial denaturation/activation step at 95 °C for 15 min, the mixture was subjected to 40 cycles of amplification (denaturation at 95 °C for 15 s, annealing and extension at 60 °C for 15 s). 

### 4.5. qPCR by OpenArray^®^ to Quantify Immune Gene Expression

To analyse the immune response against all the viruses tested (r1408-1412; r3073; r3093 and wt160), qPCR reactions based on TaqMan^™^ probes were performed using a previously designed high-performance OpenArray^®^ chip [10], which included probes for 106 transcripts related to the immune system and the reference genes. Selected transcripts, primers and TaqMan™ probes were described in Gémez-Mata et al. [10]. 

Quantitative PCRs were performed in the OpenArray^®^ system QuantStudio 12K Flex Real-Time PCR System (Applied System), sited in the Research Central Service of the University of Cordoba (Spain), using the TaqMan^™^OpenArray^®^ Real-Time PCR Master Mix kit (Applied Biosystems; Lithuania). Biological (6) and technical (1) replicates were loaded into OpenArray^®^plates.

For gene expression analysis, Ct values were obtained using the Thermo Fisher Connect^TM^ (ThermoFisher Scientific) online application, and the Relative Quantification (RQ) software. The setup was adjusted with options Benjamini-Hochberg deactivated, maximum Ct was set up at 28, AMP score was activated and HIGHSD was changed to 0.25. Fold change (FC) values were obtained by the 2^−^^ΔΔCt^ method [49]. Values were normalized with the geometric mean of the endogenous genes *rps4* and *ubq*. Organs from the control group (L-15) were used as calibrators. Genes with FC values < −1 (downregulated) or >1 (upregulated) and *p* < 0.05 were considered differentially expressed genes (DEGs).

The one-way multivariate analysis of variance (MANOVA) was used to test changes in gene expression after viral challenges. Statistical analyses were performed using SSPS v.26 (SPSS Inc., Chicago, IL, USA), *p* < 0.05 were considered significant.

## 5. Conclusions

In conclusion, the mutations in the 3’NCR of both RNA segments of the highly virulent reassortant, wt160, provoked a decrease in mortality and a different immune response, which could be partly responsible for the virulence reduction. Specifically, the infection with the RNA2-mutant provoked the most different immune response compared to the one elicited by the wild type virus, highlighting the exclusive and high upregulation of genes related to the complement system. Regarding RNA1-mutants, these viruses induced the most similar immune response to the one provoked by the wild type virus; however, some differences have been found. For instance, RNA1-mutant replication is delayed 24 h compared to the wt160 replication, which could be responsible for the lag observed in the induction of the immune response, compared to the wt160 group. Most of the genes expressed in all virus-infected groups were related to the IFN-I system, VRGs, or to the ubiquitination; however, some genes are exclusively expressed after the infection with the RNA1-mutants, such as the early induction of *pkr*, which could be also involved in the early detection of viral infection, and the downregulation of *il17rc*, which could establish a balance in the immune response to avoid an increase on the disease’s severity due to a strong immune response.

## Data Availability

The authors confirm that the data supporting the finding of this study are available within the article and its supplementary material. Raw data are available from the corresponding author, upon reasonable request.

## Supplementary Materials

The following are available online at https://www.mdpi.com/article/10.3390/pathogens10111388/s1, Table S1: Immunogen profile, expressed as fold change values (FC) ± SD (*n* = 6), in head kidney from animals inoculated with wt160, r3073, r3093 and r1408-1412. Fish were sampled at 2 and 3 days p.i.
